# The false academy: predatory publishing in science and bioethics

**DOI:** 10.1007/s11019-016-9740-3

**Published:** 2016-10-07

**Authors:** Stefan Eriksson, Gert Helgesson

**Affiliations:** 1 Centre for Research Ethics and Bioethics, Department of Public Health and Caring Sciences, Uppsala University, Uppsala, Sweden; 2 Stockholm Centre for Healthcare Ethics, Department of LIME, Karolinska Institutet, Stockholm, Sweden

**Keywords:** Predatory publishing, Publication ethics, Peer review, Bioethics

## Abstract

This paper describes and discusses the phenomenon ‘predatory publishing’, in relation to both academic journals and books, and suggests a list of characteristics by which to identify predatory journals. It also raises the question whether traditional publishing houses have accompanied rogue publishers upon this path. It is noted that bioethics as a discipline does not stand unaffected by this trend. Towards the end of the paper it is discussed what can and should be done to eliminate or reduce the effects of this development. The paper concludes that predatory publishing is a growing phenomenon that has the potential to greatly affect both bioethics and science at large. Publishing papers and books for profit, without any genuine concern for content, but with the pretence of applying authentic academic procedures of critical scrutiny, brings about a worrying erosion of trust in scientific publishing.

## Introduction: the false academy

Researchers today are under strong pressure to publish. The old slogan “Publish or perish” is probably more to the point than ever before, nowadays further underlined by the increasingly common practice of letting bibliometric data steer the allocation of faculty funding at universities, which means that apart from the individual’s career-interest in publishing, there is additional pressure to publish from one’s department.

Partly made possible by the IT revolution, an entire industry has grown up to cater to this need, mainly based on online publication, but also offering an extensive supply of conferences (Bowman 2014). The explosion of open access (OA) journals in recent years has brought with it increased opportunities to find decent journals to place academic work in. But not all actors are interested in promoting science while making their money. Rogue publishers serve their own economic interest, while creating dubious merit for scholars publishing with them.

Some scientists may wish for an exit strategy when traditional academic publishing is perceived as slow, somewhat arbitrary in its evaluation of manuscripts, and sometimes only moderately interested in one’s work (Lagoze et al. 2015). Some might search for a short-cut to getting published, while being aware that they have chosen a journal that does not live up to acceptable academic standards. Others might get fooled and publish in a non-serious journal, inadvertently subjecting themselves to criticism afterwards—what was meant to become an academic merit might become the very opposite. Those researchers are victims of what we may call the *false academy*: dubious or downright fraudulent operators who strike gold from luring the young and inexperienced (Xia et al. 2015) or from researchers trying to usurp merit as effectively and with as little effort as possible (Truth 2012).

In this paper, we describe the false academy with a particular focus on “predatory publishing”, raise the question whether traditional publishing houses have entered the same path as rogue publishers, note that bioethics is a discipline affected by this trend, and, towards the end of the paper, discuss what should be done to eliminate or reduce the effects of this development.

## Predatory journals

One obvious actor in the false academy is journals (Butler 2013). That academic journals have varying quality is widely known. But with the advent of open access, new opportunities have risen. Open access journals make their money from charging publishing fees, usually over 1000 euros per published paper, sometimes double that amount. Instead they usually do not charge anything for access, which means that the content of the journal is available without subscription. Modern publishing tools make this model both effective and highly profitable.

The great economic potential in this type of publishing has attracted all sorts of actors to start up professional journals (Schöpfel 2015), though quite a few with limited competence in high-quality academic publishing. Many of these questionable journals originate from India or Nigeria and primarily attract authors from developing countries (Xia et al. 2015). Typical cases are publishers whose only business idea is to accept as many papers as possible. This they are trying to achieve by offering swift review and comparatively low fees, while mimicking the academic ambitions of serious publishers. But there are also examples of outright fraud by “cyber criminals” who hi-jack established journals by using an exact replica of the original journal’s website online, except for the account to which the fee is sent (Beall 2016; Dadkhah and Borchardt 2016; Tin et al. 2014).

Apart from the moves from printed journals to electronic publications, and from subscription fees to publication fees, open access publication introduced another important change: in order to make money with traditional publication of subscribed journals, it was important to make sure that the journal was perceived as of reasonable quality in order for sufficiently many libraries to make the decision to pay for a subscription. With open access journals, quality no longer plays the same role—instead the important thing, in order to make money, is to find sufficiently many willing to pay to get published. Here, lack of genuine quality does not necessarily stand in the way of success, as long as appearances are kept up to some extent.

Many researchers easily recognize so-called predatory journals when an e-mail shows up offering space for an article or providing an invitation to act as editor of a special issue. For those who don’t, it can be costly to realize that they have been caught in the web of a non-serious journal and then trying to get out; for instance, to retract an article from the predatory publisher OMICS (see below for more about them) can result in over 400 dollars in administrative fees! (Beall 2015a) Sometimes it is difficult also for the experienced to distinguish the serious journals from the not-so-serious. For a long time, Bentham Science Publishers attracted a lot of scientists to send in OA papers and act as peer reviewers and on editorial boards, by the functionality and graphic design quality of their web pages and journals. Then, for several years, the stories started to build up a picture of a questionable publisher that could publish articles without peer-review and that spammed scientists with e-mails asking for papers. Eventually, in 2008, this (and other reasons) led to the creation of the Open Access Scholarly Publishers Association (http://oaspa.org/), which strives to set standards and organize the serious open access publishers (Eysenbach 2008; Grant 2009a).

## Alarming development also concerns bioethics

We have seen an alarming development in predatory publishing since then (Shen and Björk 2015) and noticed how colleagues start turning up in some of these journals. This reflects how predatory publishers increasingly target social scientists (Beall and DuBois 2016). Not surprisingly, the field in which this journal trades, bioethics, now has its own share of predatory journals. In a recent blog, we list approximately 25 predatory journals that deal in bioethics or related subjects (Eriksson and Helgesson 2016). We have probably missed some, and we expect the number to rise unless we can discourage bioethicists from lending themselves to such journals.

For those working in other fields, a good start when trying to identify the journals to avoid is a list maintained by Jeffrey Beall, an American academic and librarian who lists potentially rogue journals and publishers (available at http://scholarlyoa.com/). On the list are about nine hundred single, independent magazines, but if one adds to them all the journals published by larger publishing houses (sometimes actually more of garage operations, as Beall has revealed many times), the number of journals is over eight thousand (Shen and Björk 2015). So this industry is not insignificant!

One way to find the proper journals is to take a look at a list of recognized open access journals, the Directory of Open Access Journals (http://www.doaj.org). Obviously, there may be journals not found in either this whitelist or in Beall’s blacklist, or it might happen that a journal is incorrectly classified. Thus, researchers about to submit manuscripts also need to look into the matter themselves.

Some of the typical signs of predatory publishing include undisclosed fees, editorial boards with unknown or apparently non-existent members, flawed functionality and design of the website, and the choice of strange partners when it comes to indexing and impact calculations (Canadian Association of Research Libraries 2015; Clark 2015; Prater 2014). For a more extensive list, see Table 1.

**Table 1:** Characteristics of a predatory journal Note that the idea with this list is not to say that any journal fulfilling any of the points below is a predatory journal. But the more points on the list that apply to the journal at hand, the more sceptical you should be

The publisher is not a member of any recognized professional organisation committed to best publishing practices (like COPE or EASE)
The journal is not indexed in well-established electronic databases (like Medline or Web of Science)
The publisher claims to be a “leading publisher” even though it just got started
The journal and the publisher are unfamiliar to you and all your colleagues
The papers of the journal are of poor research quality, and may not be academic at all (for instance allowing for obvious pseudo-science)
There are fundamental errors in the titles and abstracts, or frequent and repeated typographical or factual errors throughout the published papers
The journal website is not professional
The journal website does not present an editorial board or gives insufficient detail on names and affiliations
The journal website does not reveal the journal’s editorial office location or uses an incorrect address
The publishing schedule is not clearly stated
The journal title claims a national affiliation that does not match its location (such as”American Journal of …” while being located on another continent) or includes”international” in its title while having a single-country editorial board
The journal mimics another journal title or the website of said journal
The journal provides an impact factor in spite of the fact that the journal is new (which means that the impact cannot yet be calculated)
The journal claims an unrealistically high impact based on spurious alternative impact factors (such as 7 for a bioethics journal, which is far beyond the top notation)
The journal website posts non-related or non-academic advertisements
The publisher of the journal has released an overwhelmingly large suite of new journals at one occasion or during a very short period of time
The editor in chief of the journal is editor in chief also for other journals with widely different focus
The journal includes articles (very far) outside its stated scope
The journal sends you an unsolicited invitation to submit an article for publication, while making it blatantly clear that the editor has absolutely no idea about your field of expertise
Emails from the journal editor are written in poor language, include exaggerated flattering (everyone is a leading profile in the field), and make contradictory claims (such as “You have to respond within 48 h” while later on saying “You may submit your manuscript whenever you find convenient”)
The journal charges a submission or handling fee, instead of a publication fee (which means that you have to pay even if the paper is not accepted for publication)
The types of submission/publication fees and what they amount to are not clearly stated on the journal’s website
The journal gives unrealistic promises regarding the speed of the peer review process (hinting that the journal’s peer review process is minimal or non-existent)—or boasts an equally unrealistic track-record
The journal does not describe copyright agreements clearly or demands the copyright of the paper while claiming to be an open access journal
The journal displays no strategies for how to handle misconduct, conflicts-of-interests, or secure the archiving of articles when no longer in operation

Beware that the latest trend is for predatory publishers to buy old, serious journals. In one go, they get access to former reputation, indexing, etc. For instance, the infamous “predatory” publisher OMICS bought the journal *La Prensa Medica*, which now asks bioethicists to submit anything publishable in the medical field (such as “calendars, case-reports, corrections, discussions, meeting-reports, news, orations, product reviews, hypotheses, and analyses”) for fast and efficient publication (quote from spam e-mail received). Another example of an established journal bought by OMICS is *the Electronic Journal of Biology* (http://ejbio.imedpub.com/), which can boast of being indexed by Thomson Reuters and DOAJ and thus makes it even harder to understand its true nature. Another example reported on is the journal *Experimental & Clinical Cardiology* (Spears 2014).

## Erratic peer review

A problem with these journals is their claim to have proper peer review of articles before they get accepted, although they often do not. Pre-publication peer review is broadly perceived to be the golden standard in science and although new models are gaining ground (such as post-publication review), the sub-standard journals want acceptance and international recognition and thus assure their authors that submitted articles will go through a thorough and efficient peer review. If you have high quality reviewers available, that promise might come to be, but this is seldom the case.

In an effort to reveal this state of affairs, some critically-minded researchers have been putting the journals to the test. For example, an anonymous researcher from Eastern Europe sent a fictional nonsense article to a publisher named AICIT. Very quickly the publisher wrote a fake review, accepted the article, and sent an invoice to the researcher; something that then could be revealed to all and sundry (Beall 2015b). Such disclosures have been made several times in the last few years (see e.g. Segran 2015; Stromberg 2014).

There is a more general problem with false peer review. Biomed Central (BMC) discovered in November 2014 that about fifty articles were carrying false reviews. Soon they found more cases in their portfolio of journals, scattered across different journals, authors, and topics. They suspected that there must be a number of firms behind this, selling false reviews, and therefore started an investigation. The withdrawal of articles accepted on grounds of fabricated reviews is in progress, at BMC as well as in other journals (Haug 2015), and the retracted article count is now well over three hundred.

Sometimes the authors themselves provide journals with fake peer reviewers in order to secure a positive response. A consequence of this is that some journals are now reconsidering the (fairly recent) practice of asking authors for suggested reviewers (Ferguson et al. 2014). However, predatory journals unfortunately cannot be expected to put much effort into exposing such illegitimate practices, since it is not in their interest.

## Manipulation of impact scores

Journals are usually indexed and receive impact points on the basis of how frequently their articles are cited in other articles. No questionable journals of the kind discussed here would get decent scores in such calculations if properly made, but figures can be manipulated. One way to do this is to create citation cartels, in which a number of journals enter into an agreement to quote each other’s articles to an excessive extent, i.e., by choice of the editor rather than by what the researchers find scientifically justified. Thus, they may all receive a higher impact (Bowman 2014; Sipka 2012).

Some magazines invite authors to help out with the manipulation of impact figures. For example, the *Thammasat International Journal of Science and Technology* gives the following instructions: “Please kindly give some citations related to your written article from any articles published in TIJSAT in order that the TIJSAT’s impact factor can be raised to a higher level.” (Ferguson 2015).

Another available strategy is to work with an indexing firm whose business idea is to improve journals’ official citation indexes. When the indexing service Copernicus rated a journal titled *Acta Myologica* to have superior impact to *Nature* and *Science* (with an astonishing impact of 53), Beall and others reacted on the peculiar calculation methods employed by this service, and have since exposed many more (Gutierrez et al. 2015).

One of the authors of this article was recently offered to write in a bioethics journal, but something did not feel right, so it was examined more closely. The publisher, which turned out to be the OMICS Group, described on their website how one of the benefits of publishing with them was that they are skilled at manipulating impact:

OMICS Group international journal’s [sic] are among the best open access journals in the world, set out to publish the most comprehensive, relevant and reliable information based on the current research and development on a variety of subjects. This information can be published in our peer reviewed journals with impact factors and are calculated using citations not only from research articles but also review articles (which tend to receive more citations), editorials, letters, meeting abstracts, short communications, and case reports. The inclusion of these publications provides the opportunity for editors and publishers to manipulate the ratio used to calculate the impact factor and try to increase their number rapidly. (OMICS 2015)

At least they are honest!

A peculiar way to tamper with the impact system was displayed by a company in the genetics sector that actually payed scientists if they cited their journals in their papers (Goldacre 2015). So citing scientists got some money while the company assembled citations. The higher the impact of the journal where you manage to cite the company’s papers, the more you are paid!

## Authorship for sale

Another way to get fake academic credit is by buying authorship. *Science* made a real scoop when they revealed what they called “China’s Publication Bazaar” on November 29, 2013. By mistake a journalist working at the magazine was offered to buy himself a place as author of an article that would be published in a rather reputable journal: *International Journal of Biochemistry & Cell Biology*. The journalist could play along with this scheme and follow the process from within; it turned out that four others who had received the offer actually went through with it. The actual price of getting this publication in one’s CV was the neat sum of 14,800 dollars! This was not a single, isolated event: In China there are outright paper brokers who sell access to more or less legitimate academic articles, Science’s investigation found (Hvistendahl 2013).

Not that we researchers in the West should point the finger at other parts of the world. A while ago a Canadian firm, Cloud Consulting Company, based in Toronto, advertised for thesis writers. For up to 100,000 dollars a year, the writers can devote themselves in their own home to sit and write theses for their “clients” (Coyne 2015).

The selling of authorship might occur with greater frequency in predatory publishing than in established journals, we don’t know, as predatory publishers are utterly uninterested in addressing such problems. The problem does reflect a more general trend towards profiteering on the needs and vulnerabilities that exist in a highly competitive research world (publish or perish).

## Rogue book publishers

Not only journals fool researchers. Rogue book publishers also want to make money even if what they produce does not forward science one iota. For instance, the publisher IGI Global specializes in publishing large edited collections (Bogost 2008; Weber-Wulff 2007). They press a few dozen copies that cost maybe 500 euros each. The idea seems to be that the editor of the book, a researcher craving more academic merits, gets a nice item to add to the publication list, while the publisher draws money from selling a few mandatory library copies. Ultimately the public pays the salaries of these questionable publishers, while those sections of the public truly in need of good edited collections (such as scholars from low and middle income countries who can’t afford access to many journals) stand to benefit nothing. Nor is the book likely to have any impact whatsoever on scientific development.

This market idea is just one instance of a more widespread trend called “vanity publishing”. It aims to get authors to publish at their own cost in order to give an impression of having created a solid scholarly work, although it is accepted by some “publisher” (or dressed-up printing service) for financial rather than academic reasons (Beall 2014).

## Traditional publishing houses turning to the dark side?

We suggest that there is a worrying trend that practices common among predatory publishers are becoming increasingly common also among traditional publishers. If we are right, scientific publishing is becoming increasingly compromised in quality and, thus, harder to trust.

A first example is that the familiar piracy practice of spamming researchers’ email boxes with offers to submit papers in areas they know little or nothing about (like offering a bioethicist to publish papers on radiology, gene sequencing, or whatever) seems to have spread to some legitimate journals. We have numerous times been invited to write scientific papers in journals from established publishers that focus on biology, epidemiology, etc., without any acknowledgement that our expertise lays elsewhere.

Also the practice of collecting large volumes, such as extensive anthologies, sold very expensively to libraries rather than being aimed at a broad scientific audience, seems to have spread outside predatory circles. We recently were informed by a well-known publisher that an article of ours were to be included in such a volume, which were to be printed in 175 copies that would retail for over 400 £ each. The editor was someone we had never heard of, and we had no say in the matter. While the publisher could not afford to give us any complimentary copies, the collection would be “an invaluable resource for university libraries worldwide, especially in countries where academic holdings are relatively less comprehensive” (from the e-mail informing us about the publication). Our experience does not seem like an isolated event (Anonymous academic 2015; Askey 2009; Bogost 2008; Paul 2016; Weber-Wulff 2007).

While most traditional journals have long been profit-driven, the competition from OA as well as their own forays into the world of OA have made it painfully clear that they sometimes put revenue before all else. They typically charge considerably higher fees than most predatory journals (Ahmed 2015; Bauer 2013; Butler 2016; Cofactor 2012; Graziotin et al. 2014) and frequently turn into “hybrid” journals, which is to say that they are both subscription-based and charge individual authors willing to pay for publishing open access. In adopting strategies such as these, the major publishing houses frequently draw criticism for primarily trying to maximize revenue at the expense of scientific exchange and openness (Bohannon 2014; Shen and Björk 2015; The Cost of Knowledge 2016).

A typical sign of predatory publishing is a stubborn refusal to engage with retractions, corrections or assisting in misconduct investigations. Recently some of the most prestigious journals in the medical field were criticized by Ben Goldacre on the COMPARE website for e.g. not accepting corrections to misleading articles or giving access to protocols when fraud is suspected (COMPARE 2016). Others have recently criticised one of the biggest open-access publishers, PLOS, for not providing authors with page proofs and then not publishing corrections for the resulting formatting errors (Chawla 2016).

Also, fake or lousy reviews, or editors disregarding thorough negative reviews, are not exclusive to predatory journals. When Bohannon wrote his famous fake papers and sent them to 304 publishers, Elsevier, Sage, Wolters Kluwer, and several university-based publishers were among those who accepted the papers (Bohannon 2013). Some journals count Nobel laurates among their contributors, yet reportedly accept papers after insanely fast peer review (Nature News article comments 2014).

Perhaps the most noteworthy example of reputable publishers engaging in questionable practices is the much-discussed case of Elsevier. They notably issued several journals that basically served as adverts for unnamed drug companies while appearing as peer reviewed medical journals, with no disclosure of sponsorship (Grant 2009b; Singer 2009). Elsevier is also criticized for high subscription costs that exasperate even wealthy universities such as Harvard: “We faculty do the research, write the papers, referee papers by other researchers, serve on editorial boards, all of it for free … and then we buy back the results of our labour at outrageous prices,” a Harvard library director complained to The Guardian (Sample 2012). Elsevier then in several instances charged readers for access to articles already paid for by the authors to make the articles open access (Jump 2014; Mounce 2015).

It is obvious that the greed of publishing houses may conflict with scientific goals and standards. It is troublesome if predatory publishers influence traditional publishers to increase focus on profit and feel more forgiving to quality-reducing shortcuts.

## What can we do?

What is so serious about the development we now see is that trust and confidence in academic publishing is undermined. To curb this trend, an increased awareness of the false academy must be disseminated among researchers and those who assess researchers (Tin et al. 2014; Think, Check, Submit 2016).

To date, it has primarily been individual activists and journalists (this often coincides) that have worked hard to reveal this phenomenon and to get research institutions, funders, and journals to pay attention to the problem and take action. Just to mention one example, Scientificspam.net is a niche DNSBL (which stands for a DNS-based Black List) that lists spammers targeting scientists by retrieving e-mail addresses from PubMed and similar sources, in order to get mailing lists for sending unsolicited bulk email.

A noteworthy recent institutional response is the US Federal Trade Commission charging OMICS, iMedPub and some other “predators” with having deceived researchers about their services (Federal Trade Commission 2016). This case will then be decided in court. This is very welcome, but a thorough response requires several additional actions to be taken. We propose the following actions (as a first input to the discussion):

- The forming of committees for each research field to keep track of rogue actors.
- A forum for continuous sharing of experiences of the false academy (preferably financially supported by several research-promoting government agencies).
- Further use in the research area of laws prohibiting deceptive acts or practices against consumers.
- A widespread policy among universities and research funders that individuals regularly involved in activities relating to predatory publishing should not be permitted to apply for positions, promotion, or funding.
- Other actions taken by universities, individually or jointly, in order to reduce the number of publications in predatory journals, such as blacklists.
- The allocation of funds for research on the false academy.
- Software development for fast tracking of false or dubious merits in publication lists.

Researchers all over the world are today finding new ways to share their experiences of predatory publishing practices, through blogs, commentary fields, twitter, etc. If their reports about academic publishing are only half-true, the observed behaviour threatens the scientific record by further swamping the literature with poor or misleading papers. If disguised as proper publishing, those practices will be even harder to unmask. Therefore it is due time to start spreading information on this phenomenon and to take measures to reveal the extent of shoddy practices and false merit. We welcome further bioethics community debate and the reporting of experiences, both in this journal and at our blog on where to publish and not to publish in bioethics (Eriksson and Helgesson 2016).

## Conclusions

Predatory publishing is a growing phenomenon that affects bioethics as well as science at large. The publishing of papers and books for profit, without any genuine concern for content, but with the pretence of applying authentic academic procedures of critical scrutiny, brings about an erosion of trust in scientific publishing. These concerns relate to so-called predatory journals and book publishers, and possibly also to more traditional publishers. The gravity of the problem calls for action. We have described some present endeavours and suggested further desirable actions. Maybe a greater change is required in the longer run, where commercial and career interests are forced to take a backseat and publishing again becomes primarily a matter of furthering scholarly exchange and scientific development (Poss et al. 2014; Parsons 2016). Even though there are interesting initiatives, such as Ubiquity Press (http://www.ubiquitypress.com/), it remains to be seen how that can be accomplished on a greater scale.^1^

## Footnotes

1 Some of the examples of questionable practices used in this article were first collected for an opinion piece by SE that was published in the Swedish journals Svensk Onkologi and Svensk Kirurgi in 2015. Permission for reuse of that material in this updated and heavily expanded paper has kindly been granted.
